# Synthetic microbial communities for engineering climate‐smart biofertilizers

**DOI:** 10.1002/imt2.70140

**Published:** 2026-06-11

**Authors:** Yan Liu, Yue Chen, Yanxue Yu, Che Ok Jeon, Mohammad Bahram, Junfeng Zhai, Hailei Wei, Fuqiang Wang, Xiaofeng Cao, Baolei Jia

**Affiliations:** ^1^ Xianghu Laboratory Hangzhou China; ^2^ Horticulture Research Institute Zhejiang Academy of Agricultural Sciences Hangzhou China; ^3^ Institute of Plant Quarantine Chinese Academy of Quality and Inspection & Testing Beijing China; ^4^ Department of Life Science Chung‐Ang University Seoul Republic of Korea; ^5^ Department of Agroecology, Faculty of Technical Sciences Aarhus University Slagelse Denmark; ^6^ Department of Ecology Swedish University of Agricultural Sciences Uppsala Sweden; ^7^ Institute of Agricultural Resources and Regional Planning Chinese Academy of Agricultural Sciences Beijing China; ^8^ Hainan Seed Industry Laboratory Sanya China; ^9^ Institute of Genetics and Developmental Biology Chinese Academy of Sciences Beijing China

## Abstract

Global agricultural productivity is increasingly destabilized by climate change—driven droughts, floods, extreme heat, and severe storms. Although the climate‐smart agriculture (CSA) framework addresses these challenges, implementation has focused mainly on plant genetics and agronomic inputs, leaving the adaptive potential of the crop microbiome underexplored. Here, we examine the agricultural use of synthetic microbial communities (SynComs) through the “crop holobiont” concept, in which plants and their associated microbiota function as an integrated, responsive system rather than through plant genomes alone. Pioneer plants in extreme environments may serve as reservoirs of stress‐adapted microbes and provide a strategic toolkit for advancing CSA. SynComs assembled from these microbes can act not only as nutrient suppliers but also as dynamic physiological modulators that enhance crop phenotypic plasticity under climatic stress. We propose a roadmap for crop microbiology that integrates synthetic ecological engineering, with broad implications for CSA.

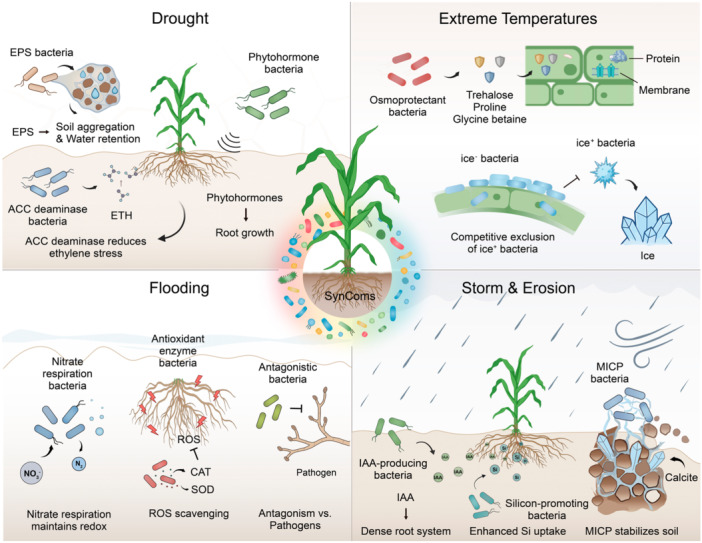

Global agriculture faces three major challenges: feeding a growing population, adapting to climate change, and reducing greenhouse gas emissions. Accelerating climate change, including rising temperatures, elevated atmospheric CO_2_, and more frequent extreme events, is already causing yield declines in major staple crops and is projected to drive severe and uneven production losses worldwide (Figure S1) [1]. In response to these pressures, a climate‐smart agriculture (CSA) framework was conceived by the Food and Agriculture Organization of the United Nations to transform agricultural systems and ensure food security in an increasingly volatile climate [2]. CSA is built on three pillars: sustainably increasing productivity, enhancing climate resilience, and reducing greenhouse gas emissions.

However, current CSA strategies largely remain embedded in conventional crop breeding and input‐management approaches. Traditional crop improvement has mainly focused on genetically encoded traits, such as plant height and branching, to maximize yields under stable environments. Although these approaches have achieved major successes, their effectiveness is declining under increasingly unpredictable climate stresses. Importantly, both breeding and agronomy have largely overlooked the plant‐associated microbiome, including microorganisms inhabiting plant tissues, surfaces, and the rhizosphere, often referred to as the plant's “second genome.”

Recently, the concept of the “crop holobiont” has been proposed, framing the crop as a composite organism in which the plant and its associated microorganisms interact to collectively regulate growth, development, nutrient acquisition, and stress responses. Microbial interactions can rapidly modulate plant acclimation to changing environments through diverse regulatory pathways [3]. Thus, the holobiont concept suggests that the function of plants extends beyond static genetic determinism, with microbial partners instead being indispensable regulators of plant resilience and productivity.

We propose that the future of CSA depends on shifting from plant‐centric models to the crop holobiont paradigm. To operationalize this shift, synthetic microbial communities (SynComs)—purposefully assembled consortia designed to optimize natural microbiome functions—have emerged as powerful, precision tools [4]. In this perspective, we discuss CSA within the crop holobiont framework, explore how SynComs from pioneer plants in extreme environments can function as climate‐smart biofertilizers, and evaluate potential inoculation strategies.

## RETHINKING CLIMATE‐SMART AGRICULTURE WITHIN THE CROP HOLOBIONT PARADIGM

Traditional agricultural breeding strategies have focused on genetically determined morphological traits, such as plant height, leaf angle, and branching patterns, to maximize yield under specific conditions. Although this genome‐centered approach produced high‐yield wheat and semidwarf rice cultivars, it largely overlooked the regulatory role of the plant microbiome.

### From plant microbiomes to functional holobiont

The plant microbiome functions as an external regulatory system that influences plant development and stress responses under climate change. By integrating this functionality and given that most plant‐associated microbes originate from soil, the soil‐plant system shapes a holobiont, an evolutionarily and functionally integrated unit comprising the host plant and its associated microbial community [5]. In this system, microbial partners actively regulate crop resilience and ecosystem functions, including nutrient cycling, soil aggregation, and plant health. These microbiome‐mediated functions support the three pillars of CSA by improving productivity, enhancing stress resilience, and reducing dependence on synthetic fertilizers.

**Figure 1:**
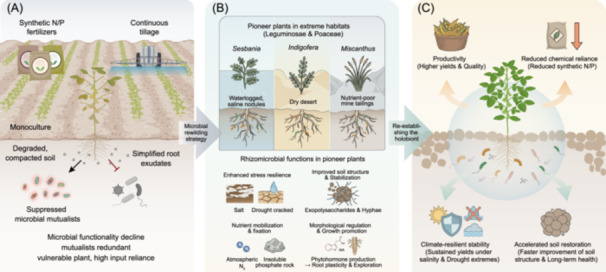
Leveraging pioneer plant‐associated microbes for microbial rewilding. (A) Conventional intensification and domestication have caused a decline in microbial functionality, rendered mutualists redundant, and simplified root regulatory pathways. Microbial rewilding addresses reduced functionality by recruiting beneficial consortia to restore lost ecosystem functions. (B) Pioneer plants, particularly members of the Leguminosae and Poaceae families (such as *Sesbania*, *Indigofera*, and *Miscanthus*), represent critical and underexplored reservoirs of stress‐adapted and functionally potent microbes. (C) By reconceptualizing the crop as a dynamic holobiont, pioneer‐plant‐derived microbes simultaneously increase productivity (yield), resilience (salinity/drought tolerance), and mitigation potential (reduced synthetic N/P reliance), thereby fulfilling the three core pillars of climate‐smart agriculture (CSA).

Evidence suggests that microbial functions may have declined in modern agricultural ecosystems because of excessive use of fertilizer, continuous tilling, and domestication syndrome (Figure 1A). Over‐fertilization increases nitrogen and phosphorus inputs. Long‐term tillage accelerates organic matter mineralization. Domestication has made modern crops dependent on high‐fertility conditions, reinforcing nutrient‐rich rhizosphere environments. High nutrient availability can render microbial mutualists functionally redundant and inhibit microbial colonization. Moreover, modern high‐yield crops may produce simplified root exudates, further reducing beneficial plant–microbe interactions [6].

### Extreme environments as reservoirs for stress‐adapted microbes

One strategy is microbial rewilding: reintroducing ancestral microbial communities to restore lost ecosystem function (Figure 1B,C). Microbial rewilding reduces synthetic chemical use and mitigates climate impacts by improving plant health. Currently, the microbiota of crop wild relatives is used for rewilding [7]. However, surviving extreme climates requires adaptive traits beyond historical baselines, so pioneer plant microbiomes in extreme environments are underexplored reservoirs. Pioneer plants colonize nutrient‐poor habitats (e.g., deserts, saline‐alkali land) and catalyze succession. Unlike woody pioneers on centennial timescales, Leguminosae and Poaceae pioneers offer annual‐cycle benefits: increasing nitrogen fertility and improving soil structure [8].

The genera *Sesbania* and *Indigofera* (both Leguminosae) and *Miscanthus* (Poaceae) contain species that colonize resource‐limited soil, with high root‑to‑shoot ratios and deep, plastic root systems [9]. *Sesbania*'s ability to form nitrogen‑fixing nodules on roots and stems is intertwined with its microbial community. *Rhizobium huautlense* facilitates nodulation in *Sesbania herbacea* under both flooded and nonflooded conditions, thus enabling growth in waterlogged environments [10]. Microbes from non‑legume pioneers also show biofertilizer potential. The pioneer grass *Miscanthus sinensis*, thriving in nutrient‑poor mine tailings, harbors *Rhizobium* sp. G‑14 and *Pseudomonas* sp. Y‑5. Co‐inoculation of these two strains into *Bidens pilosa* increased colonization, nitrogen fixation, and growth [11].

In summary, pioneer plants serve as important microbial reservoirs, which may be useful in the process of microbial rewilding. Harnessing microbial resources associated with pioneer plants offers a promising pathway to develop climate‐resilient crops and enhance the adaptive capacity of agricultural systems in an increasingly volatile environment.

## SYNCOMS AS CLIMATE BUFFERS: MECHANISMS AND COLONIZATION STRATEGIES

The inherent complexity of natural soil microbiomes has resulted in a conceptual shift in agricultural microbiology from microbial inoculants with a narrow scope to the use of designed SynComs. Unlike previous trial‐and‐error approaches that introduced beneficial microbes individually, multispecies SynComs are designed to provide defined, reproducible functions tailored to hosts and environments [4]. Rational SynCom design generally follows two complementary strategies, namely top‐down approaches that distill functionally coherent minimal consortia from complex native microbiomes, and bottom‐up approaches that assemble defined communities from well‐characterized isolates. Increasingly, these strategies are guided by computational modeling and multi‐omics analyses, including genome‐scale metabolic models, Lotka–Volterra models, reverse ecology, and machine learning, to predict microbial interactions, enhance functional complementarity, and improve community stability under variable field conditions (Figure S2; See Supplementary data). One of the main goals of next‐generation SynCom design is to develop microbial communities that function as living climate buffers, dynamically modulating plant physiology against climate‐induced stress. Accordingly, rationally designed SynComs aim to enhance plant tolerance to drought, flooding, extreme temperatures, and physical disturbance through crop holobiont engineering, thereby supporting the adaptation pillar of CSA.

### Mechanisms of SynCom‐mediated climate resilience

**Figure 2:**
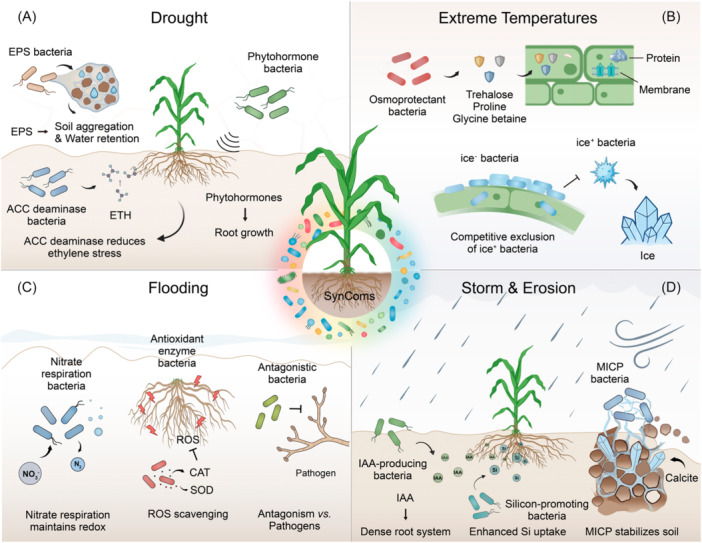
Proposed SynComs improve crop resilience to drought, extreme temperatures, flooding, and storms to support CSA. (A) SynComs mitigate drought stress by secreting EPS, modulating phytohormone levels, and delaying plant senescence via ACC deaminase activity, among other synergistic tolerance mechanisms. (B) SynComs alleviate heat stress by supplying chemical chaperones that stabilize membranes and proteins. During frost events, ice^−^ mutants competitively exclude ice‐nucleating bacterial strains, thereby providing biological antifreeze activity to maintain cellular integrity. (C) SynComs ameliorate flooding effects and anoxic stress through nitrate‐respiring members that maintain rhizosphere redox potential during inundation. Microbial enzymes neutralize bursts of ROS, and antagonistic taxa form a biotic shield against opportunistic root‐rot pathogens. (D) SynComs attenuate the effects of physical disturbances such as storms via MICP and silicon‐mediated cell wall reinforcement. Additionally, auxin production optimizes root anchoring, and rapid biofilm formation protects wound sites from pathogen invasion. ACC, 1‐aminocyclopropane‐1‐carboxylic acid; CAT, Catalase; CSA, climate‐smart agriculture; EPS, exopolysaccharides; ETH, Ethylene; IAA, indole‐3‐acetic acid; ice^+^, ice‐promoting bacteria; ice^−^, ice‐nucleation‐deficient bacteria; MICP, microbially induced calcite precipitation; ROS, reactive oxygen species; SynComs, synthetic microbial communities; Si, silicon; SOD, Superoxide dismutase.

Among these climate stressors, drought is affecting crop production globally. SynComs can increase drought tolerance through multiple complementary mechanisms [12], including reducing stress‐induced senescence via microbial ACC (1‐aminocyclopropane‐1‐carboxylic acid) deaminase production, improving water retention through osmoprotectants, and enhancing water acquisition through phytohormone production and root architecture modulation (Figure 2A). Microbial exopolysaccharides promote soil aggregation and increase water‐holding capacity in the rhizosphere. Consistent with this, SynComs derived from xerophytic plant rhizospheres improve crop drought tolerance through changes in stomatal density and xylem architecture, linking natural microbial adaptation to engineered climate resilience.

In addition to drought, extreme temperatures further constrain crop productivity. Heat stress causes protein denaturation and membrane destabilization, which SynComs can alleviate by supplying osmoprotective metabolites such as trehalose, proline, and glycine betaine (Figure 2B) [13]. These substances act as chemical chaperones (small molecules that assist protein folding and stabilize membrane structures) that stabilize the protein and membrane structures of microbial cells and plant hosts and improve thermotolerance at the plant‐microbe holobiont level. Under cold conditions, SynComs incorporating ice‐nucleation‐deficient (ice^−^) *Pseudomonas syringae* mutants can suppress frost damage by competing with ice‐promoting bacteria on leaves [14].

Flooding and waterlogging create hypoxic conditions that damage crops through oxygen deprivation and toxic metabolite accumulation. SynComs containing nitrate‐respiring bacteria can improve root survival by using nitrate as an alternative electron acceptor, thereby maintaining rhizosphere redox balance (Figure 2C). This process preserves a favorable redox environment in the rhizosphere and limits the accumulation of reduced compounds. Severe tissue damage often occurs during post‐flood reoxygenation when reactive oxygen species overwhelm the antioxidant defenses of plants. SynComs capable of generating catalase and superoxide dismutase can neutralize oxidative bursts before plant roots become irreversibly injured [15]. In addition, antagonistic microbial taxa may help suppress root‐rot pathogens favored by flooded soils.

Physical disturbances associated with storms, including soil erosion and root damage, may also be mitigated through SynComs (Figure 2D). For example, physical effects may be reduced through microbially induced calcite precipitation [16]. Mechanistically, this process relies on SynComs harboring ureolytic bacteria that hydrolyze urea, elevate local pH, and promote calcium carbonate precipitation, ultimately inducing mild rhizosphere calcification that reinforces soil structural integrity and prevents erosion during intense rainfall. Similarly, SynComs may facilitate silicon uptake, strengthen cell walls, and increase resistance to lodging and breakage [17]. To limit uprooting, SynComs can be engineered to influence root architecture. Microbially produced auxins, such as indole‐3‐acetic acid, stimulate the formation of lateral roots and the proliferation of root hairs, which can increase root surface area and the ability to bind soil [18]. Lastly, after physical injury, plants become vulnerable to opportunistic pathogens entering through wounds. SynComs can be designed to detect signals that are associated with wounding, such as jasmonate, and to populate damaged tissues rapidly. By forming protective biofilms, SynComs may promote plant recovery from physical disturbance.

In summary, SynComs can mitigate drought, extreme temperatures, flooding, and storm‐related damage through complementary microbial mechanisms, acting as versatile buffers against climate stressors. We propose that ideal SynComs should integrate functional guilds specialized in hydrological regulation, thermal protection, oxidative stress mitigation, physical defense, and root architecture modulation. However, field application remains challenging because of strain incompatibility, environmental instability, and trade‐offs among stress‐mitigation functions. Overall, eco‐engineered SynComs provide a promising strategy for supporting resilient CSA systems under increasing climate variability.

### Strategies for robust field colonization

Achieving these climate‑buffering functions critically depends on whether the introduced SynCom establishes in the field. Engineering the crop holobiont requires the introduced SynCom to establish in competitive native soils; otherwise, even an optimal consortium may fail. As soil microbiota is fiercely resistant to invasion, a simplistic probiotic approach is insufficient for effective SynCom design. Colonization strategies must consider invasion ecology principles, such as exploiting priority effects where early colonizers shape final community assembly. SynComs can be incorporated onto seed surfaces using microbial‐compatible polymers or alginate‐based coatings; upon germination, they rapidly colonize the nascent root surface before native microbes occupy this niche [19]. Competitive fitness can be enhanced by bacterial antagonism traits: Type VI secretion systems (T6SSs) inject toxic effectors into competitors, improving persistence in crowded settings. Finally, functional redundancy is essential for community stability; designing SynComs around functional guilds rather than individual species ensures performance of taxonomically distinct members, safeguarding community integrity if specific strains are lost to predation, phage infection, or competitive exclusion.

Consequently, advanced delivery strategies are essential for SynCom success. One promising “seed‐and‐feed” approach coencapsulates SynComs with prebiotic substrates metabolized by members, enhancing microbial fitness and competitive advantage in the rhizosphere [20]. An alternative uses succession‐based seed coatings for staggered release of distinct strains over time. SynComs can be translated from laboratory to variable field environments via advanced design and delivery systems. Although SynCom introduction may transiently affect native microbiomes, functionally redundant consortia integrate without displacing keystone taxa. Recent metatranscriptomic evidence confirms stress‐adaptive gene expression in SynCom‑inoculated crops under field stress. Pilot field trials using pioneer‑plant microbes have shown yield benefits, though large‑scale and long‑term safety challenges remain. When appropriately deployed, SynComs act as living buffers that enhance crop resilience under climate change.

## IMPLICATIONS AND FUTURE DIRECTIONS

The integrated CSA framework addresses food security and adaptation to climate change. However, to transform CSA from a conceptual framework into a scalable solution, we need to integrate it in the precision agriculture using modern biological technologies. In this context, SynCom‐based biofertilizers represent a critical step in this transition. Herein, we term climate‐smart biofertilizers as rationally designed SynComs that act as dynamic physiological modulators enhancing crop phenotypic plasticity against climate‐induced stresses through complementary mechanisms such as osmoprotectant production, root architecture modulation, and rhizosphere engineering, beyond conventional nutrient provisioning, thereby supporting agricultural adaptation to increasing climatic volatility within the CSA framework.

Advances in plant microbiome research and ecological theory now enable rational design of functionally optimized microbial consortia. Their development relies on integrating high‐throughput screening, multi‐omics, mechanistic modeling, and ecological principles. However, further work is needed to optimize SynCom performance and assess long‐term sustainability under field conditions. Future efforts should focus on understanding SynCom–host–soil interactions, developing scalable delivery systems, and evaluating ecological impacts and field persistence. Integration with existing agricultural practices, including breeding, precision agriculture, fertilizers, and pesticides, is also essential for large‐scale CSA implementation.

## AUTHOR CONTRIBUTIONS

**Yan Liu:** Visualization, writing—review and editing, data curation; **Yue Chen:** Writing—review and editing; **Yanxue Yu:** Writing—review and editing; **Che Ok Jeon:** Writing—review and editing; **Mohammad Bahram:** Writing—review and editing; **Junfeng Zhai:** Writing—review and editing; **Hailei Wei:** Writing—review and editing; **Fuqiang Wang:** Writing—review and editing; **Xiaofeng Cao:** Writing—review and editing; **Baolei Jia:** Writing—original draft, validation, funding acquisition.

## CONFLICT OF INTEREST STATEMENT

The authors declare no conflict of interest.

## ETHICS STATEMENT

No animals or humans were involved in this study.

## Supporting information


**Figure S1:** Historical climatic extremes and projected impacts on agricultural productivity in the future.
**Figure S2:** Design strategies for synthetic microbial communities (SynComs).

## Data Availability

The data that support the findings of this study are available in the supplementary material of this article. The data and scripts used are saved in GitHub https://github.com/yanliu2023/iMeta-commentary/. Supplementary materials (data, figures, graphical abstract, slides, videos, Chinese translated version, and updated materials) may be found in the online DOI or iMeta Science http://www.imeta.science/.
