# Supplementary material for: Synthetic microbial communities for engineering climate‐smart biofertilizers
Source: Imeta. 2026 Jun 11;5(3):e70140. doi: 10.1002/imt2.70140 (PMC13377407; doi:10.1002/imt2.70140)
Supplement: Supplementary file 1 — Figure S1: Historical climatic extremes and projected impacts on agricultural productivity in the future. Figure S2: Design strategies for synthetic microbial communities (SynComs). [file IMT2-5-e70140-s001.docx]

Supporting Information to

**Synthetic microbial communities for engineering climate-smart biofertilizers**

**Running title:** SynComs for climate-smart biofertilizers

Yan Liu^1#^, Yue Chen^1, 2#^, Yanxue Yu^3#^, Che ok Jeon^4^, Mohammad Bahram^5, 6^, Junfeng Zhai^3*^, Hailei Wei^7*^, Fuqiang Wang^8*^, Xiaofeng Cao^9*^, Baolei Jia^1*^

^1^Xianghu Laboratory, Hangzhou 311200, China

^2^Horticulture Research Institute, Zhejiang Academy of Agricultural Sciences, Hangzhou 310021, China

^3^Institute of Plant Quarantine, Chinese Academy of Quality and Inspection & Testing, Beijing 100176, China

^4^Department of Life Science, Chung-Ang University, Seoul 06974, Republic of Korea

^5^Department of Agroecology, Faculty of Technical Sciences, Aarhus University, Slagelse 4200, Denmark

^6^Department of Ecology, Swedish University of Agricultural Sciences, Uppsala 756 51, Sweden

^7^Institute of Agricultural Resources and Regional Planning, Chinese Academy of Agricultural Sciences, Beijing 100081, China

^8^Hainan Seed Industry Laboratory, Sanya 572025, China

^9^Institute of Genetics and Developmental Biology, Chinese Academy of Sciences, Beijing 100101, China

^#^These authors contributed equally: Yan Liu, Yue Chen, Yanxue Yu

^*^Correspondence: zhaijf@caqit.org.cn (Junfeng Zhai), [weihailei@caas.cn](mailto:weihailei@caas.cn) (Hailei Wei), wangfuqiang@yazhoulab.com (Fuqiang Wang), xfcao@genetics.ac.cn (Xiaofeng Cao), jiabaolei@xhlab.ac.cn (Baolei Jia)

**Supplementary data**

**Global climate change and its impacts on crop yields**

Rapid climate change during the past half-century has produced an increase in the global mean surface temperature of 0.20 °C per decade since 1982, marking a sharp acceleration from the historical rate of 0.06 °C per decade recorded since 1850. Concurrently, the annual growth rate of atmospheric CO_2_ accelerated from 0.8 ppm in the 1960s to an unprecedented 2.6 ppm between 2015 and 2024, driving the global mean concentration to 422.7 ppm in 2024. High atmospheric CO_2_ concentrations can increase photosynthesis in C3 crops such as wheat and rice, but these potential gains are often negated by heat stress and a limited water supply.

Rainfall regimes have become increasingly erratic. Despite a small overall increase in global precipitation, there is strong regional heterogeneity. Subtropical areas, such as the Mediterranean Basin and Southern Africa, have experienced continual drying, while areas at higher latitudes are generally becoming wetter. This has increased the occurrence and severity of extreme climate events. More frequently, severe heatwaves are occurring over most land areas, but intense rainfall and flooding have also become more frequent. Such extremes constitute immediate climatic threats to crop production and may cause catastrophic yield losses within a single growing season.

Historical records capture the scale of these changes in precipitation and temperature (Figure S1A). Annual global drought events jumped from 0.64 during 1900–1949 to 16.76 in the 2000–2025 period, a staggering > 26-fold increase, and China consistently recorded the highest incidence. The frequency of extreme temperature events has increased even more dramatically, from 0.04 to 168.3 events per year, with India experiencing an overwhelming share of these events. The number of floods has increased from 1.08 per year to 22.72 per year, and the number of major storms has increased from 3.18 to approximately 110 per year, with the United States experiencing the greatest increase in these events. Since 2000, the intensity of these extremes has exceeded the records for events during the first half of the 20th century, which indicates that the climate system has become increasingly erratic and extreme.

The impact of climate change on staple crops is already visible. A combination of global models, local experiments, and statistical regressions revealed that for each 1 °C increase in global mean temperatures, average yields would decrease by approximately 6% for wheat, 3.2% for rice, 7.4% for maize, and 3.1% for soybeans. Even accounting for the benefits of CO_2_ fertilization and adaptive interventions, simulations and historical analyses still suggest that each increase of 1 °C will cause a 4.1–6.4% decline in global wheat yields. According to a study based on a nonlinear econometric framework covering 12,658 subnational regions, six staple crops are projected to experience severe but spatially uneven yield losses under high-emission trajectories by the end of this century (Figure S1B). Maize production in the United States and China may decline by ~40%, soybean production in the United States may decline by ~50%, and wheat production in major regions may decline by 15–40%. Declines in rice production in Sub-Saharan Africa and Central Asia will exceed 50%. The production of cassava, which is important to low-income populations, is expected to decline by approximately 40% in Sub-Saharan Africa. Increases in production at higher latitudes will be too limited to compensate for these declines.

Given the severe and uneven yield losses projected for major staple crops under climate change as outlined above, CSA (climate-smart agriculture) must evolve beyond conventional crop improvement paradigms. This reality underscores the growing importance of harnessing plant microbiomes as first responders to climate stress, positioning them as increasingly vital allies in strengthening the climate resilience of agricultural systems.

**Rational design of synthetic microbial communities**

The inherent complexity of natural soil microbiomes has resulted in a conceptual shift in agricultural microbiology from microbial rewilding (which relies on microbial inoculants with a narrow scope) to the use of thoughtfully assembled SynComs (synthetic microbial communities). Unlike previous trial-and-error approaches that have introduced beneficial microbes individually, multispecies SynComs are designed to provide defined and reproducible functions that are tailored to hosts and environments. Currently, there are two complementary strategies to SynCom development and construction: the top-down and bottom-up approaches (Figure S2).

The top-down approach to SynCom construction begins with a complex microbiome that exhibits a particular property or function. From that naturally complex community, the aim is to distill a minimal community that optimally performs the desired function in a laboratory setting. High-throughput sequencing and ecological network analyses are employed to identify keystone taxa or core community members that are statistically associated with healthy phenotypes in plants. In contrast, the bottom-up approach involves building de novo communities from well-characterized microbial isolates, which enables hypothesis-driven experimentation and engineering of novel functional traits. For example, extensive isolate collections from *Arabidopsis* revealed that leaf- and root-derived SynComs preferentially shape their respective native microbiomes, illustrating the ecological principle of habitat-specific colonization fitness.

Despite the availability of these approaches for SynCom construction, the combinatorial complexity of potential microbial interactions remains a design challenge. As the number of microbial strains increases, the potential pairwise and higher-order interactions grow exponentially, making it difficult to predict how specific combinations will behave under environmental fluctuations, particularly when disruption of a single keystone interaction can collapse overall community functionality. Therefore, computational modeling has become essential for SynCom design. Specifically, genome-scale metabolic models can be used to predict metabolic complementarity, cross-feeding, and competition among community members. These insights facilitate the rational assembly of consortia with enhanced stability and functionality. Employing the reverse ecology facilitates the identification of species that are important for community performance and enables the separation of large natural microbiomes into minimal functional synthetic systems. Machine-learning analysis of multiomics data can support the identification of microbial interactions and keystone taxa that can be used to achieve desirable effects, such as pathogen suppression, which then allows for data-driven optimization of SynCom composition. Together, these computational approaches transform SynCom design from a trial-and-error process into a predictive discipline, enabling researchers to anticipate emergent community properties and engineer minimal consortia that reliably function under fluctuating field conditions.

Despite these advances, major bottlenecks remain, including poor model predictability under field conditions, low colonization efficiency, and unassessed ecological safety. Future improvements should focus on iterative model and experiment refinement, synthetic biology tools for field persistence, and standardized long-term risk assessment frameworks.





**Figure S1 Historical climatic extremes and projected impacts on agricultural productivity in the future**. (A) Frequencies of droughts, extreme temperatures, floods, and storms since 1900. Data were sourced from the EM-DAT (The Emergency Events Database) international disaster database. (B) Projected global yield losses by 2100 under high-emission scenarios.


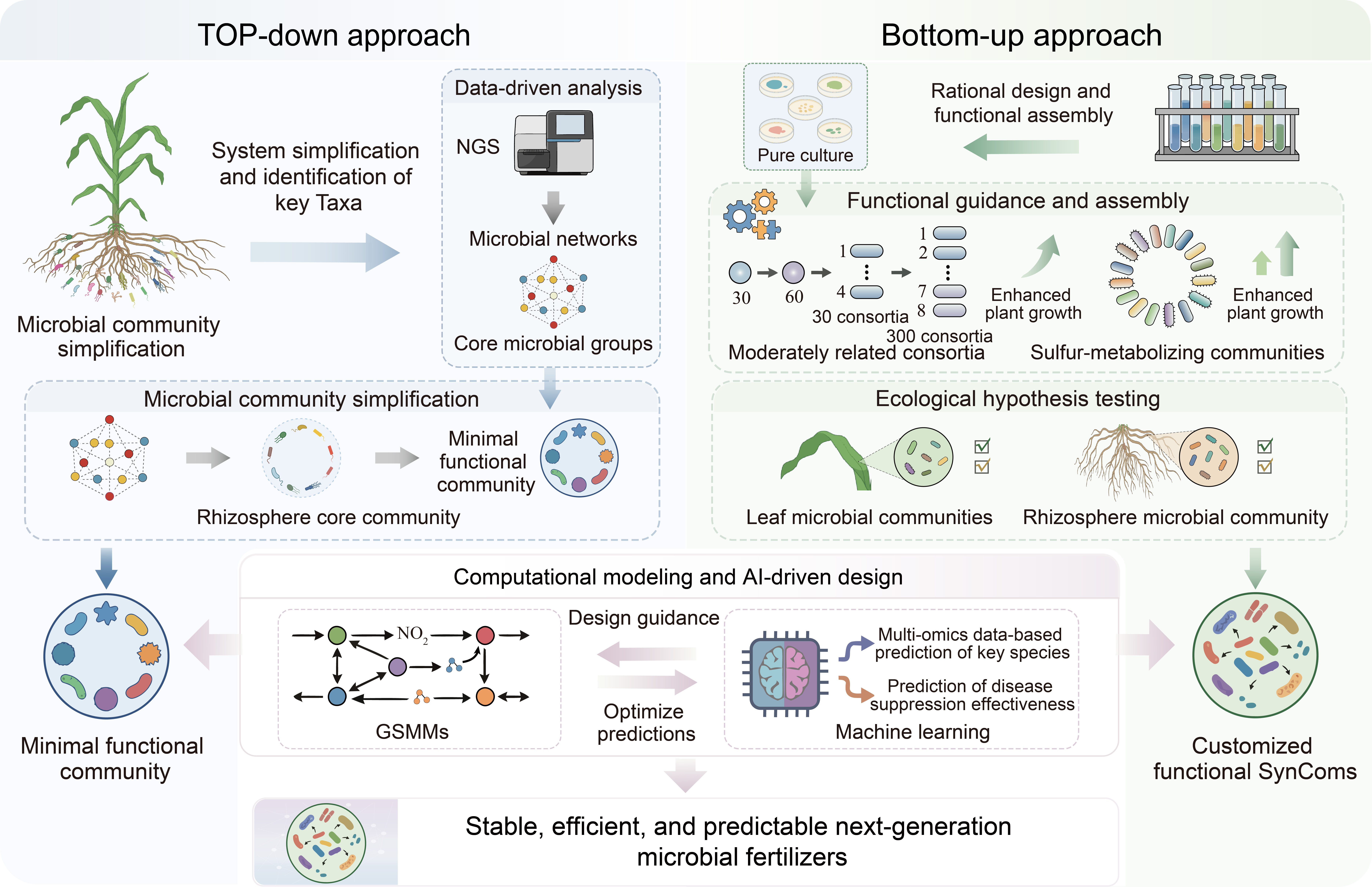


**Figure** **S2** **Design strategies for SynComs.** A top-down approach distills complex natural microbiomes into minimal functional units through network-based analysis. Conversely, a bottom-up approach uses well-characterized microbial isolates and assembles a community guided by ecological principles. The integration of computational modeling enables the rational engineering of SynComs with predictable metabolic complementarity and stability by simulating interspecies interactions. Abbreviation: SynComs, Synthetic microbial communities; NGS, Next-generation sequencing; GSMMs, Genome-scale metabolic models.
